# A nationwide cohort study on the risk of ADHD in children with amblyopia mediated by fine motor skill impairment in East Asia

**DOI:** 10.1038/s41598-022-10845-1

**Published:** 2022-04-28

**Authors:** Myungjin Kim, Seungwon Lee, Jung Eun Lee, Ju Hee Kim, Eun Kyo Ha, Manyong Han, Helen Lew

**Affiliations:** 1 Department of Ophthalmology, CHA Bundang Medical Center, CHA University School of Medicine, 59, Yatap-ro, Bundang-gu, Seongnam-si, Gyeonggi-do 13496 Republic of Korea; 2 Department of Data Science, Sejong University College of Software Convergence, Seoul, Republic of Korea; 3 Department of Data Science, Sungkyunkwan University School of Medicine, Seoul, Republic of Korea; 4 Department of Pediatrics, Hallym University Kangdong Sacred Heart Hospital, Seoul, Republic of Korea; 5 Department of Pediatrics, Hallym University Kangnam Sacred Heart Hospital, Seoul, Republic of Korea; 6 Department of Pediatrics, CHA Bundang Medical Center, CHA University School of Medicine, 59, Yatap-ro, Bundang-gu, Seongnam-si, Gyeonggi-do 13496 Republic of Korea

**Keywords:** Medical research, Signs and symptoms

## Abstract

This national administrative investigation of Republic of Korea compared the risk of attention deficit hyperactivity disorder (ADHD) and autism spectrum disorders(ASD) in preschool amblyopic children and identified factors that possibly mediate this association. After propensity score (PS) matching, 7762 amblyopic children and 31,030 non-amblyopic children were included. Amblyopia was associated with ADHD (aOR:1.687; 95% CI 1.444, 1.970) but not with ASD (aOR: 0.591; 95% CI 0.341, 1.026). Fine motor skill impairment was a mediating factor in association of amblyopia with ADHD, accounting for 4.2% (95% CI 1.7, 8.0). In conclusion, amblyopic children have a greater risk of ADHD, and deficits in fine motor skills mediate this association. We suggest increased attention given to fine motor skill underdevelopment in amblyopic children to prevent the development of ADHD.

## Introduction

Amblyopia is a common cause of vision impairment whose prevalence rate is approximately 2% in preschool children and 3% in adults^1–3^. Joly and Franko^4^ defined amblyopia as the reduction of best-corrected visual acuity to less than 6/9 in Snellen optotype or at least two-line difference in LogMAR optotype between the eyes. This reduced visual acuity is not caused by structural abnormalities of the eye, and typically cannot be corrected by glasses alone.

Some studies reported an association between visual deficits and reduced psychosocial development^5–7^, motor skill development^8,9^, and neurodevelopmental diseases, such as attention-deficit hyperactivity disorder (ADHD) and autism spectrum disease (ASD)^10–14^. Although previous studies reported a linkage of amblyopia as a single factor with delayed child development or with neurodevelopmental diseases, the mediation factors among amblyopia, neurodevelopmental diseases, and delayed child development such as motor dysfunction, are not well known.

Recent studies demonstrated that the abnormal perceptual experience caused by impaired vision in these patients can influence the microstructural integrity of white matter and lead to cortical changes^15,16^. The frontal aslant tract (FAT) in white matter of the brain, which connects the inferior frontal gyrus with the supplementary and presupplementary motor areas, is regarded to be involved in visuomotor processing. Altered FAT might be involved in the neuromechanism underlying defective upper limb coordination in patients with amblyopia resulting in fine motor impairment^17^. Also laterality of the microstructural properties of the FAT was associated with greater attention and executive function problems, providing new potential structural biomarker to assess ADHD or associated executive dysfunction during development^18^.

In this study,. we investigated whether developmental delay in children with amblyopia, including reduced motor skills, was associated with neurodevelopmental diseases such as ADHD and ASD. A better understanding the association between motor dysfunction and neurodevelopmental diseases in children with amblyopia may help to improve treatments and help to correct motor dysfunctions during early childhood. We used the nationwide database of the Republic of Korea to analyze the effect of amblyopia on the risk of ADHD and ASD and to identify factors that possibly mediated this association.

## Results

### Participants

We first examined the basic sociodemographic and clinical characteristics of children in the amblyopia and non-amblyopia groups (Table 1 and Supplementary Table 1). Before propensity score(PS) matching, the following standardized differences were greater than 5%: sex; residence at birth; prematurity; birth weight; hospital utilization within 4 months; disorders related to maternal, gestation and fetal factors; disorders related to respiratory and cardiovascular factors; disorders related to transitory endocrine and metabolic factors; and chromosomal abnormalities. After PS matching, the two groups were balanced in all demographic and clinical baseline characteristics. We also determined the prevalence of amblyopia according to age at diagnosis (Supplementary Fig. 1).

**Table 1:** Basic characteristics of children in the main cohort. Data are presented as n (%) or mean, SD.

Sociodemographic characteristics	Unmatched data (N = 401,852)			PS matched data (N = 38,792)		
	Amblyopia (N = 8100)	Non-amblyopia (N = 393,752)	Standardized difference (%)	Amblyopia (N = 7762)	Non-amblyopia (N = 31,030)	Standardized difference (%)
**Sex**						
Boy	3936 (48.6)	203,626 (51.7)	6.2	3772 (48.6)	14,864 (47.9)	1.4
Girl	4164 (51.4)	190,126 (48.3)		3990 (51.4)	16,166 (52.1)	
**Residence at birth**						
Seoul	2156 (26.6)	96,340 (24.5)	6.2	2117 (27.3)	8611 (27.8)	1.3
Metropolitan	1962 (24.2)	92,430 (23.5)		1901 (24.5)	7623 (24.6)	
Urban	3069 (37.9)	155,421 (39.5)		2956 (38.1)	11,731 (37.8)	
Rural	840 (10.4)	46,052 (11.7)		785 (10.1)	3065 (9.9)	
**Birth year**						
2008	3952 (48.8)	187,097 (47.5)	2.5	3806 (49.0)	15,240 (49.1)	0.1
2009	4148 (51.2)	206,655 (52.5)		3956 (51.0)	15,790 (50.9)	
**Income quintile**						
1 (lowest)	561 (6.9)	30,248 (7.7)	3.6	546 (7.0)	2162 (7.0)	0.7
2	1154 (14.3)	57,634 (14.6)		1132 (14.6)	4391 (14.2)	
3	2174 (26.8)	105,724 (26.9)		2160 (33.5)	8709 (28.1)	
4	2605 (32.2)	125,176 (31.8)		2599 (33.5)	10,466 (33.7)	
5 (highest)	1327 (16.4)	61,200 (15.5)		1325 (17.1)	5302 (17.1)	
**Prematurity**						
No	7429 (91.7)	370,179 (94.0)	9.6	7119 (91.7)	28,546 (92.0)	0.9
Yes	671 (8.3)	23,015 (5.9)		643 (8.3)	2484 (8.0)	
Birth weight (kg)	3.16, 0.47	3.20, 0.44	9.7	3.15, 0.47	3.15, 0.46	1.2

### Statistical analysis

Between-group differences in baseline characteristics were compared using standardized differences in unmatched and PS matched samples. A differences greater than 10% was considered meaningful. PS matched risk ratios and 95% CIs were obtained using modified Poisson regression, and PS matched risk differences and 95% CIs were obtained using a binomial regression model with an identity link function. The mediation analysis focused on the identification of factors that mediate the association of amblyopia with neurologic diseases. To increase the robustness of the results, analyses were adjusted for the same covariates used in PS matching. This analysis was implemented using the difference in coefficients, in which the change in the logistic regression coefficient β associated with amblyopia (adjusted for the covariates of sex, residence at birth, birth year, income quintile, prematurity, birth weight, hospital utilization within 4 months, perinatal conditions, and congenital/chromosomal abnormalities) was assessed with subsequent adjustment for potential mediating factors. Two-tailed P values less than 0.05 were interpreted as significant. All analyses were conducted using SAS version 9.4 (SAS Institute Inc, Cary, NC). The mediation analysis was performed in three stages.

### Stage 1: amblyopia in children with different neurodevelopmental characteristics

The association of amblyopia with different neurodevelopmental characteristics was first assessed by calculating the least-squares estimate of the mean risk according to the presence of amblyopia. Dichotomous factors were coded as 0 or 1, so the mean for each variable was equivalent to the proportion with the characteristic. The least-squares means and 95% CIs were reported for children with and without amblyopia, and the differences and 95% CIs of these differences were calculated.

### Stage 2: mediating effects of neurodevelopmental characteristics in the association of amblyopia with ADHD

The effect of neurodevelopmental factors in mediating the association of amblyopia with ADHD was determined by calculating odds ratios (ORs) and using logistic regression to adjust for covariates (sex, residence at birth, birth year, income quintile, prematurity, birth weight, hospital utilization within 4 months, perinatal conditions, and congenital/chromosomal abnormalities). All ORs were presented according to the presence or absence of each potential mediating factor. The proportion of the population with ADHD among those with *vs.* without each factor was calculated using the same logistic model, and absolute differences were calculated as the differences in these proportions. The 95% CIs for proportions and differences were calculated using bootstrap methods with 1000 replicates.

### Stage 3: magnitude of the mediating effects identified in stage 2

The magnitude of the mediating effects identified in Stage 2 was assessed as the difference in the β coefficient for two logistic models used to predict ADHD after adjusting for sex, residence at birth, birth year, income quintile, prematurity, birth weight, hospital utilization within 4 months, perinatal conditions, and congenital/chromosomal abnormalities. The 95% CIs for the differences in the β coefficients between the two models were calculated using bootstrap methods with 1000 replicates.

### Risk of the neurodevelopmental diseases in the amblyopia and non-amblyopia groups

We then assessed the risk of ADHD and ASD in the amblyopia and non-amblyopia groups (Table 2). This analysis of matched data in the main cohort indicated a statistically significant association of amblyopia with ADHD (OR: 1.687; 95% CI 1.444, 1.970) but not with ASD (OR: 0.591; 95% CI 0.341, 1.026). Based on these results, we performed mediation analysis for ADHD in boys and girls (see below). The prevalence of ADHD was 2.9% (221/7762) in the amblyopia group and 1.7% (516/31,030) in the non-amblyopia group. The prevalence of ASD was 0.2% (14/7762) in the amblyopia group and 0.3% (91/31,030) in the non-amblyopia group.

**Table 2:** Risk for ADHD and ASD in the amblyopia and non-amblyopia groups (PS matched data).

	Amblyopia (N = 7762)	Non-amblyopia (N = 31,030)	OR (95% CI)	P value	Adjusted OR (95% CI)	P value
ADHD	221 (2.9)	516 (1.7)	1.712 (1.466, 2.000)	< 0.001	1.687 (1.444, 1.970)	< 0.001
ASD	14 (0.2)	91 (0.3)	0.615 (0.351, 1.079)	0.090	0.591 (0.341, 1.026)	0.061

### Risk of delayed development and mediating effect of ADHD in boys

**Figure 1:**
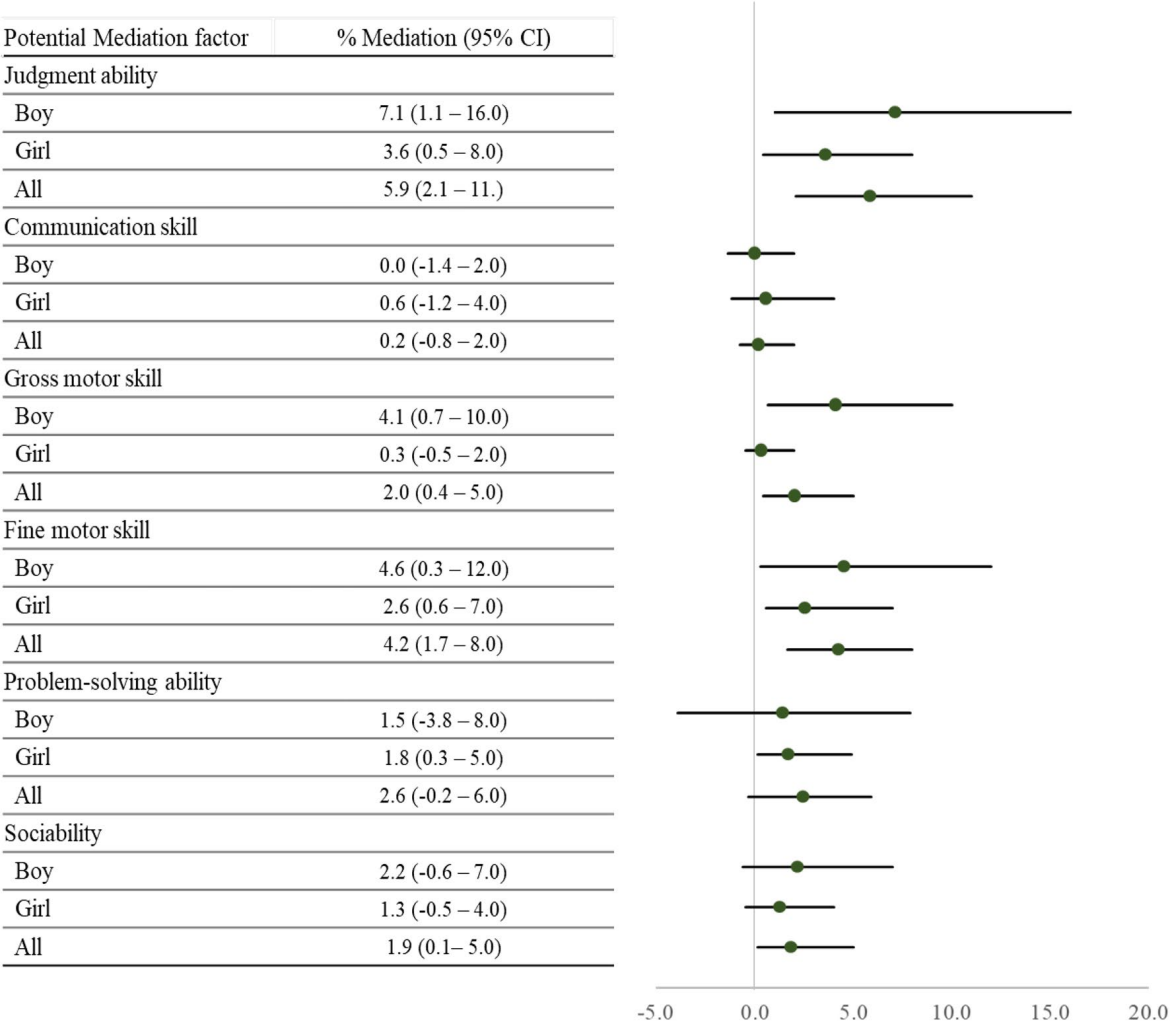
Strengths of the mediating effects of different factors in increasing the risk for ADHD in children with amblyopia (Left: %; Right: aOR and 95% CI).

We analyzed the result of Korean-Ages and Stages Questionnaire (K-ASQ) assessing the judgment ability, communication skills, gross motor skills, fine motor skills, problem-solving ability, and sociability which was considered as potentialmediating factors in case of underdevelopment, between amblyopia and ADHD in boys (Table 3). The least-squares mean for fine motor skills impairment was 3.12% (95% CI 2.83, 3.41) for boys in the non-amblyopia group and 3.96% (95% CI 3.39, 4.53) for those in the amblyopia group, corresponding to a mean difference of − 0.84% (95% CI − 1.49, − 0.20). Mediation analysis indicated a significant association between fine motor skill impairment and ADHD (adjusted OR: 1.38; 95% CI 1.10, 1.66). The incidence proportion of ADHD was 4.45% (95% CI 2.74, 6.17) in boys without fine motor skill impairment and 12.01% (95% CI 9.84, 14.18) in those with fine motor skill impairment, corresponding to an absolute risk difference of 7.56% (95% CI 6.14, 8.98). Thus, fine motor skill impairment was a mediating factor, in that it increased the incidence of ADHD in boys by 4.6% (95% CI 0.31, 12.0) (Fig. 1).

**Table 3:** Effects of different factors in mediating the association between amblyopia and ADHD in boys.

Potential mediating factor	Sample size		Least-squares me an(95% CI)		Difference between amblyopia and non-amblyopia children	Adjusted odds ratio (95% CI)	Adjusted incidence proportion (95% CI)		Absolute risk difference in incidence (95% CI)
	Amblyopia	Non-amblyopia	Amblyopia	Non-amblyopia			Factor absent (95% CI)	Factor present (95% CI)	
Judgement ability	3493	13,886	5.88 (5.17, 6.57)	4.75 (4.39, 5.10)	− 1.13 (− 1.90, − 0.34)	1.52 (1.29, 1.74)	3.84 (2.11, 5.56)	12.62 (10.6, 14.6)	8.87 (7.62, 9.95)
Communication skills	3693	14,551	0.46 (0.24, 0.67)	0.44 (0.34, 0.55)	− 0.01 (− 0.25, 0.23)	0.93 (0.14, 1.72)	4.88 (3.16, 6.60)	9.87 (5.75, 14.0)	4.99 (1.20, 8.78)
Gross motor skills	3677	14,535	2.02 (1.63, 2.41)	1.37 (1.18, 1.57)	− 0.65 (− 1.09, − 0.21)	1.45 (1.06, 1.84)	4.59 (2.88, 6.31)	13.16 (10.5, 15.8)	8.57 (6.49, 10.65)
Fine motor skills	3641	14,398	3.96 (3.39, 4.53)	3.12 (2.83, 3.41)	− 0.84 (− 1.49, − 0.20)	1.38 (1.10, 1.66)	4.45 (2.74, 6.17)	12.01 (9.84, 14.2)	7.56 (6.14, 8.98)
Problem-solving ability	3631	14,328	2.37 (1.89, 2.83)	2.17 (1.93, 2.40)	− 0.2 (− 0.73, 0.33)	1.57 (1.26, 1.88)	4.58 (2.87, 6.30)	14.58 (12.2, 17.0)	9.99 (8.27, 11.72)
Sociability	3632	14,366	2.34 (1.89, 2.79)	1.89 (1.66, 2.11)	− 0.45 (− 0.96, 0.05)	1.20 (0.83, 1.56)	6.24 (4.09, 8.39)	11.37 (8.91, 13.8)	6.51 (4.69, 8.32)

### Risk of delayed development and mediating effect of ADHD in girls

We performed the same analysis for girls (Table 4). The least-squares mean for the fine motor skill impairment was 1.24% (95% CI 1.06, 1.41) for girls in the non-amblyopia group and 1.94% (95% CI 1.57, 2.30) for those in the amblyopia group, corresponding to a mean difference of − 0.70% (95% CI − 1.10, − 0.29). Mediation analysis indicated an association between the fine motor skill impairment and ADHD (adjusted OR: 1.73; 95% CI 1.05, 2.40). The incidence proportion of ADHD was 0.98% (95% CI 0.14, 1.83) in girls without fine motor skill impairment and 3.97% (95% CI 2.66, 5.27) in those with fine motor skill impairment, corresponding to an absolute risk difference of 2.98% (95% CI 1.97, 3.99). Thus, fine motor skill impairment was a mediating factor, in that it increased the incidence of ADHD in girls by 2.6% (95% CI 0.60, 7.00) (Fig. 1).

**Table 4:** Effects of different factors in mediating the association between amblyopia and ADHD in girls.

Potential mediating factor	Sample size		Least-squares mean (95% CI)		Difference between amblyopia and non-amblyopia children	Adjusted Odds Ratio (95% CI)	Adjusted Incidence Proportion (95% CI)		Absolute risk difference in incidence (95% CI)
	Amblyopia	Non-amblyopia	Amblyopia	Non-amblyopia			Factor Absent (95% CI)	Factor Present (95% CI)	
Judgement ability	3818	15,519	2.97 (2.49, 3.46)	2.30 (2.06, 2.54)	− 0.68 (− 1.21, − 0.14)	2.01 (1.53, 2.49)	0.85 (0.01, 1.69)	4.75 (3.64, 5.85)	3.90 (3.14, 4.66)
Communication skill	3915	15,880	0.28 (0.13, 0.43)	0.21 (0.13, 0.28)	− 0.07 (− 0.24, 0.09)	2.22 (1.02, 3.42)	1.00 (0.15, 1.84)	6.99 (4.37, 9.60)	5.99 (3.50, 8.48)
Gross motor skill	3890	15,835	1.50 (1.15, 1.86)	1.24 (1.06, 1.41)	− 0.26 (− 0.66, 0.13)	1.05 (0.14, 1.97)	1.00 (0.16, 1.85)	2.26 (0.94, 3.58)	1.25 (0.21, 2.30)
Fine motor skill	3847	15,708	1.94 (1.57, 2.30)	1.24 (1.06, 1.41)	− 0.70 (− 1.10, − 0.29)	1.73 (1.05, 2.40)	0.98 (0.14, 1.83)	3.97 (2.66, 5.27)	2.98 (1.97, 3.99)
Problem-solving ability	3866	15,748	2.09 (1.70, 2.48)	1.43 (1.24, 1.62)	− 0.66 (− 1.09, 0.22)	1.48 (0.74, 2.13)	0.98 (0.14, 1.82)	3.16 (1.91, 4.41)	2.18 (1.24, 3.13)
Sociability	3632	14,366	2.34 (1.89, 2.79)	1.89 (1.66, 2.11)	− 0.42 (− 0.81, 0.03)	1.48 (0.74, 2.21)	0.97 (0.13, 1.81)	3.40 (2.07, 4.72)	2.42 (1.37, 3.47)

## Discussion

This study demonstrated that children with amblyopia had a higher risk of ADHD than those without amblyopia, and that children with amblyopia also had delayed childhood development characterized by lower cognitive abilities (judgement ability, problem-solving ability), social skills (communication skills, sociability), and motor skills(gross motor skills, fine motor skills). Our mediation analysis suggested that fine motor skill impairment mediated part of the relationship between amblyopia and ADHD in boys and girls.

Previous studies also reported that ADHD was associated with ocular abnormalities, including amblyopia, especially when diagnosed at a younger age^11,12^. This supports the hypothesis that impaired visual perception during childhood caused by amblyopia can hamper childhood development and make children more vulnerable to neurological disorders such as ADHD. The currently accepted hypothesis is that amblyopia arises from a mismatch of the images from the two eyes. In other words, the brain favors information from one eye and suppresses information from the other eye, possibly because of cortical neural loss in the region associated with the unfavored eye. A brain imaging study showed that amblyopia was associated with neural loss in V1, known as the primary visual cortex^4,15^. Other studies suggested that visual cortex abnormalities might be related to impairments in early-stage attentional mechanisms and therefore increase the likelihood of ADHD^19^. The present study provides evidence that vision problems caused by amblyopia are associated with underdevelopment of attentional mechanisms, which may lead to delayed development of fine motor skills and contribute to ADHD. The limited ophthalmic experiences of children with amblyopia is likely to interfere with the normal development of ophthalmic function derived from the visual cortex, and eventually lead to inadequate development of regions in the brain related to attention. Amblyopia should therefore be considered a risk factor for ADHD.

Children who have amblyopia have delayed development and a higher risk of ADHD, and deficits in the development of fine motor skills appears to mediate part of the increased risk for ADHD in these children. Our study thus suggests that increased attention should be devoted to the underdevelopment of fine motor skills in children with amblyopia to reduce the risk of ADHD. In other words, the lack of normal visual development in children with amblyopia may hinder their development of motor skills and cognitive functions that are essential for normal brain development. This may manifest as altered development of the ophthalmic system and general health.

The key strength of this study is that it had a population-based design with a relatively large sample of preschool children, meaning that our results can be considered generalizable. However, this study had some limitations. First, the diagnoses of amblyopia and other comorbid diseases were entirely based on ICD-10 codes. Second, the visual acuity examination was based on home screening or screening conducted in public healthcare centers, and it was not possible to obtain accurate information about best-corrected vision acuity, which is considered a reliable indicator of potential visual function. For this reason, our database had no information on the severity of amblyopia, and we could not investigate different subtypes of amblyopia as surrogates for different levels of visual performance.

Nevertheless, the findings of our study demonstrated an increased risk of neuronal developmental problems in children with amblyopia. This suggests that suboptimal development of visual acuity may be associated with other general health problems that are related to development and other neurological disorders. Additional longitudinal research should therefore examine the functional correlation between the severity of visual impairment and other systemic disorders.

The primary target in this population-based cohort study of data from a nationwide health insurance research database were children with amblyopia. The prevalence of amblyopia was 1.8% in this sample of 917,707 Korean preschool children, comparable to other studies of preschool children^8,9,11,12^. Some previous studies reported an association between visual deficits and reduced visuomotor skills or psychosocial development^5–7^, and other studies suggested a relationship between ophthalmic disorders and some specific diseases^11,12,20^. These findings are consistent with our results that the risk of ADHD was higher in children with amblyopia and that impaired development of fine motor skills mediated some of this effect.

## Methods

### Study design and setting

This retrospective population-based cohort study was conducted using data from the National Health Insurance Service (NHIS) of Korea, which has mandatory participation for all residents. Data were from the National Investigation of Birth Cohort in Korea study 2008 (NICKs-2008), which has information on children born in 2008 and 2009^21^. A total of 917,707 children were included (469,248 born in 2008 and 448,459 born in 2009).

The NHIS maintains health records regarding healthcare utilization, prescriptions, and national health screening programs for all residents of Korea. The National Health Screening Program for Infants and Children (NHSPIC) database includes information on developmental screening, which was implemented from second round to the seventh round when children were 9–71 months-old. NHIS health records up to December 2017 were analyzed, and included the results of the fifth, sixth, and the seventh NHSPIC of the study population. The use of de-identified individual data for research purposes was authorized under the current National Health Insurance Act. The protocol of this study was reviewed and approved by the Institutional Review Board of the Korea National Institute for Bioethics Policy (P01-201603-21-005). All study conduct adhered to the tenets of the Declaration of Helsinki. Informed consents were obtained from all participants of the study.

### Data sources

Demographic characteristics, health care utilization, and outcome data of all participants were obtained from the NICKs-2008 database. The developmental screening tool used for the NHSPIC was the K-ASQ, a parent-based screening test. The K-ASQ evaluates six domains in child development: judgment ability, communication skill, gross motor skills, fine motor skills, problem-solving ability, and sociability. The diagnosis of ADHD and ASD was based on codes from the International Classification of Diseases 10^th^ revision (ICD-10). ADHD was diagnosed when a child had ICD-10 code F90.0A, F90.0B, or F90.0C at least twice, with an interval between codes of more than 1 year^22^; ASD was diagnosed when a child had ICD-10 code F84.0, F84.1, or F84.9 at least twice, with an interval between codes of more than 6 months^23^. The clinical diagnoses of ADHD and ASD in the registration database has high validity^22–24^.

### Study population

The main cohort consisted of children who were enrolled in NICKs-2008, participated the first round of the NHSPIC, participated in at least one round from the fifth to seventh rounds of the NHSPIC, and had properly recorded K-ASQ results through the NHSPIC. Children who died were excluded. A total of 401,852 children met the inclusion and exclusion criteria, 8100 were diagnosed with amblyopia and the other 393,752 children did not have amblyopia. Amblyopia was identified by ICD-10 codes of H53.00 (Unspecified amblyopia), H53.01 (Deprivation amblyopia), H53.02 (Refractive amblyopia), or H53.03 (Strabismic amblyopia), with evaluation by ophthalmologists based on the NHSPIC and visual acuity examination.

**Figure 2:**
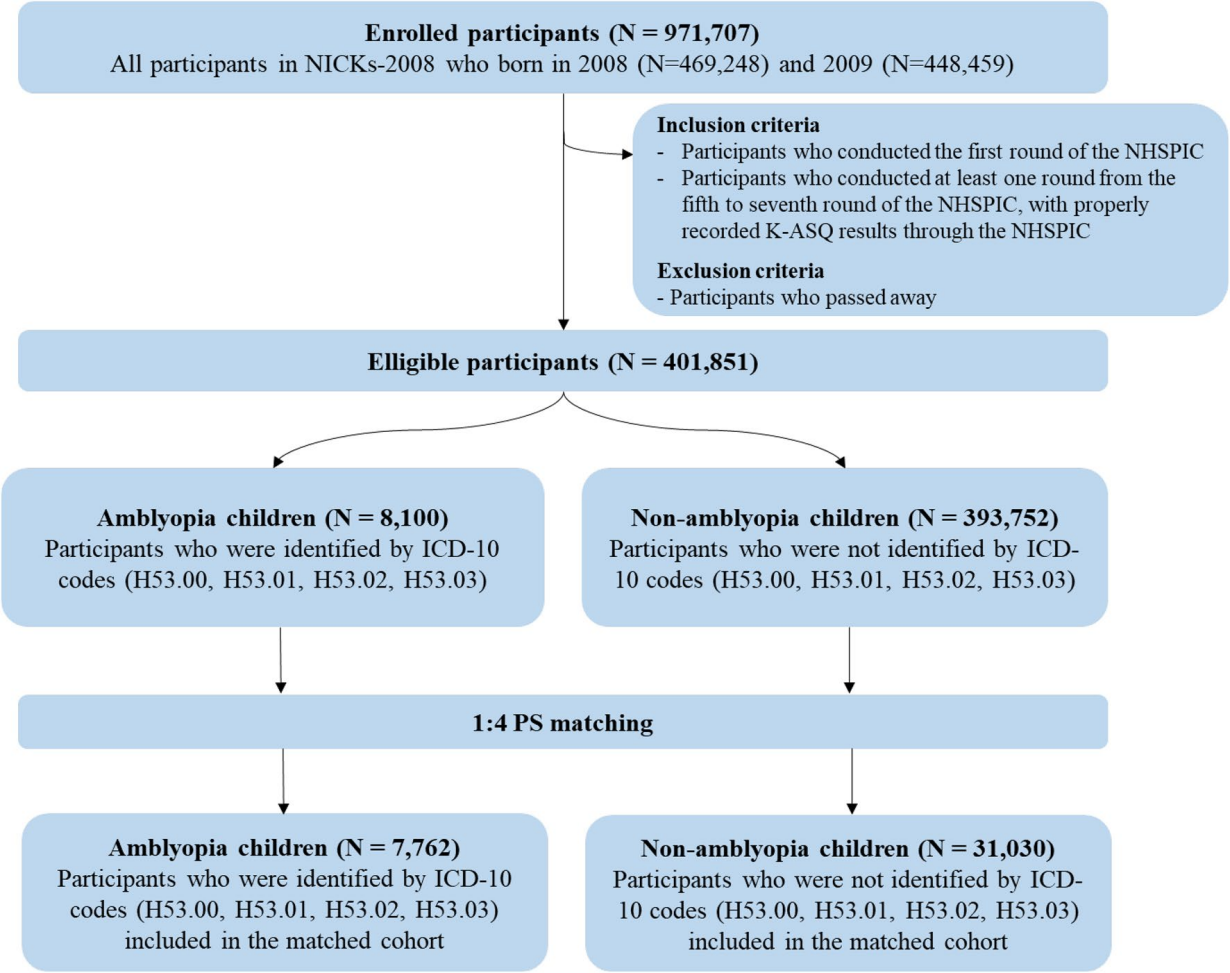
Disposition of children in the study cohort.

Propensity score (PS) matching was performed using appropriate covariates (Table 1 and Supplementary Table 1) to reduce potential confounders and balance the baseline covariates of the amblyopia and non-amblyopia groups. The demographic variables included sex, residence at birth, birth year, income quintile (based on insurance premium), prematurity, birth weight, hospital utilization within 4 months, perinatal conditions, and congenital/chromosomal abnormalities. Perinatal conditions and congenital/chromosomal abnormalities were evaluated by ICD-10 codes (P and Q codes, respectively) from the NHIS. After 1:4 PS matching, 7762 children were assigned to the amblyopia group, and 31,030 children to non-amblyopia group (Fig. 2).

### Outcomes

All outcomes were prespecified. The primary outcome was the risk of ADHD and ASD and the secondary outcome was the risk of delayed development. Developmental problems were assessed using K-ASQ data collected during the fifth to seventh rounds of the NHSPIC, when children were 42–72 months-old. Mediation analysis was performed separately for boys and girls to evaluate the effect of developmental problems in mediating the association of amblyopia with neurologic diseases. Mediation analysis estimates the proportion of an association (in this study, the association of amblyopia with neurologic diseases) that can be attributed to potential mediating factors (in this study, development problems).

## Supplementary Information

Supplementary Information.
