# Supplementary material for: A nationwide cohort study on the risk of ADHD in children with amblyopia mediated by fine motor skill impairment in East Asia
Source: Sci Rep. 2022 Apr 28;12:6932. doi: 10.1038/s41598-022-10845-1 (PMC9051132; doi:10.1038/s41598-022-10845-1)
Supplement: Supplementary file 1 — Supplementary Information. [file 41598_2022_10845_MOESM1_ESM.docx]

**Supplementary Table 1. Basic clinical characteristics of children in the main cohort.**

| Clinical  Characteristics | Observed Data (N = 401,852) | | |  | PS Matched Data (N = 38,792) | | |
| --- | --- | --- | --- | --- | --- | --- | --- |
|  | Amblyopia  (N = 8100) | Non-amblyopia  (N = 393,752) | Standardized Difference, % |  | Amblyopia  (N = 7762) | Non-amblyopia  (N = 31,030) | Standardized Difference, % |
| Hospital utilization within 4 months, N (%) | | | | | | | |
| No | 7694 (95.0) | 380,093 (96.5) | 7.7 |  | 7372 (95.0) | 29,687 (95.7) | 3.2 |
| Yes | 406 (5.0) | 13,659 (3.5) |  |  | 390 (5.0) | 1343 (4.3) |  |
| Conditions (ICD-10) during the perinatal period, N (%) | | | | | | | |
| Maternal factors | 284 (3.5) | 10,531 (2.7) | 5.0 |  | 276 (3.6) | 994 (3.2) | 1.9 |
| Disorders related to length of gestation and fetal period | 423 (5.2) | 13,880 (3.5) | 8.4 |  | 407 (5.2) | 1462 (4.7) | 2.3 |
| Birth trauma | 102 (1.3) | 3869 (1.0) | 2.9 |  | 100 (1.3) | 347 (1.1) | 1.6 |
| Respiratory and cardiovascular disorder specific to the perinatal period | 623 (7.7) | 24,390 (6.2) | 6.0 |  | 602 (7.8) | 2272 (7.3) | 1.5 |
| Infections specific to the perinatal period | 1343 (16.6) | 60,714 (15.4) | 3.1 |  | 1285 (16.6) | 4981 (16.1) | 1.3 |
| Hemorrhagic and hematological disorders of fetus and newborn | 2882 (35.6) | 133,800 (34.0) | 3.4 |  | 2767 (35.7) | 10,769 (34.7) | 1.9 |
| Transitory endocrine and metabolic disorders | 488 (6.0) | 18,221 (4.6) | 6.3 |  | 470 (6.1) | 1651 (5.3) | 3.1 |
| Digestive system disorders of fetus and newborn | 264 (3.3) | 12,287 (3.1) | 0.9 |  | 255 (3.3) | 881 (2.8) | 2.5 |
| Conditions involving the integument and temperature regulation | 296 (3.7) | 14,870 (3.8) | 1.2 |  | 276 (3.6) | 1003 (3.2) | 1.7 |
| Congenital malformations, deformations in the perinatal period | 588 (7.3) | 25,146 (6.4) | 3.0 |  | 568 (7.3) | 2245 (7.2) | 0.2 |
| Chromosomal abnormality | 1072 (13.2) | 42,302 (10.7) | 7.8 |  | 1031 (13.3) | 4169 (13.4) | 0.6 |


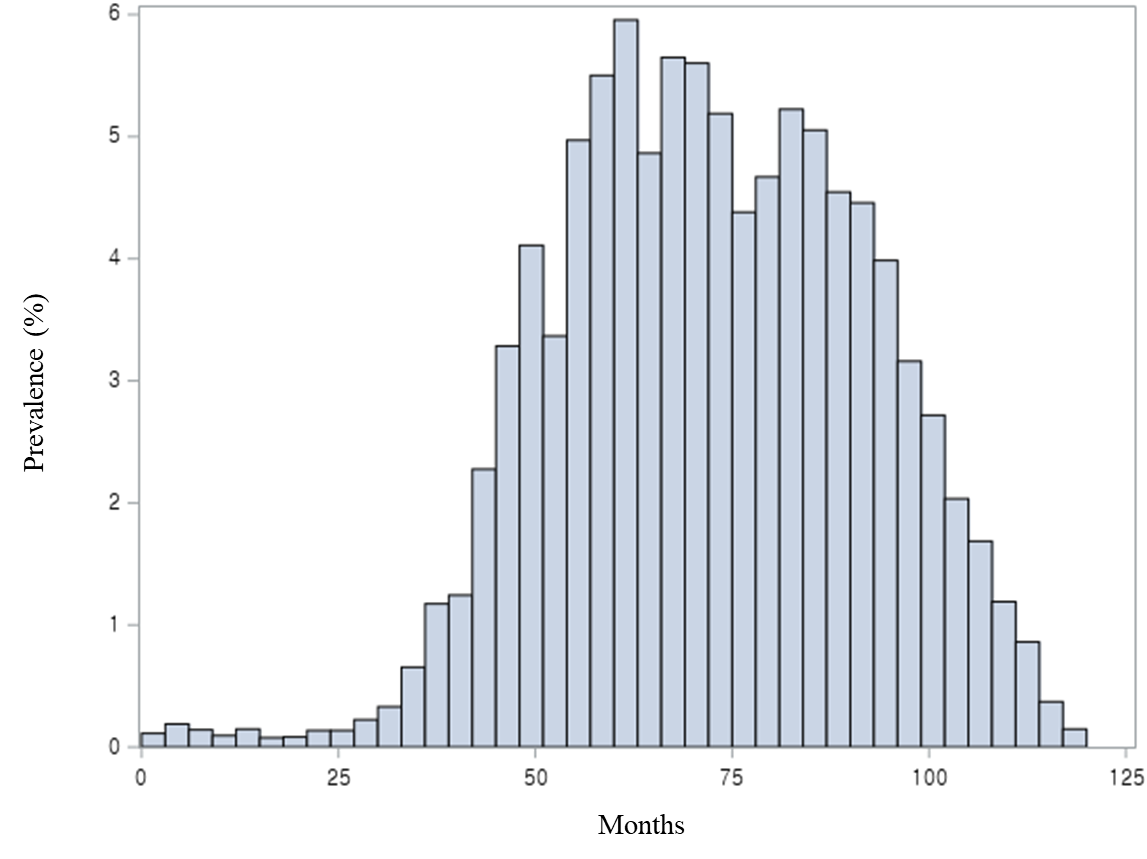


**Supplementary Figure 1. Prevalence of amblyopia according to age at diagnosis .**
